# Epidemiology of *Schistosoma haematobium* infection and microhaematuria among schoolchildren in a setting of sustained mass drug administration in Banfora, Burkina Faso

**DOI:** 10.1016/j.parepi.2025.e00470

**Published:** 2025-12-09

**Authors:** Mamoudou Cissé, Alamissa Soulama, Constant Sirima, Arthur D. Djibougou, Souleymane Gnissi, Seydou Nakanabo-Diallo, Muhammed Afolabi, Issaka Zongo

**Affiliations:** a Institut Supérieur des Sciences de la Santé, Université Nazi BONI, 01 1091 Bobo-Dioulasso, Burkina Faso; b Laboratoire de Recherche, Centre MURAZ, 01 BP390 Bobo-Dioulasso, Burkina Faso; c London School of Hygiene & Tropical Medicine, Keppel Street, WC1E 7HT London, United Kingdom; d Institut de Recherche en Sciences de Santé, Centre National de la Recherche Scientifique et Technologique, 01 BP 545 Bobo-Dioulasso, Burkina Faso

**Keywords:** Urogenital schistosomiasis, Microhaematuria, Epidemiology, Schoolchildren, Mass drug administration, Burkina Faso

## Abstract

**Background:**

Limited evidence exists regarding the current epidemiology of *Schistosoma (S.) haematobium* infection following decades of mass drug administration implementation in many areas of Burkina Faso, including the Cascades region, which is predominantly a riverine community with a high risk of schistosomiasis. This study aimed to determine the prevalence and correlates of *S. haematobium* infection and microhaematuria among schoolchildren in the municipality of Banfora, southwestern Burkina Faso.

**Methods:**

An analytical cross-sectional study was conducted in November 2024 among schoolchildren aged 5–15 years in the Banfora municipality. Sociodemographic and water contact data were collected using a pre-tested structured questionnaire. Each consenting child provided a freshly voided urine sample, which was examined for the presence of *S. haematobium* eggs and microhaematuria using the urine filtration technique and urine multistix reagent test strips, respectively. Correlates of *S. haematobium* infection and microhaematuria were determined using multivariable logistic regression.

**Results:**

The mean age of the children was 8.79 ± 2.22 years. Of the 300 school children tested, 11 (3.67 %) were found to be infected with *S. haematobium* eggs. The geometric mean intensity of *S. haematobium* infection was 14.94 eggs/10 mL of urine (95 % CI: 4.96–44.98), and 27.27 % of the infected participants (3/11) had a heavy infection. The prevalence of microhaematuria was 13.33 % (40/300). Being a boy was the main risk factor for *S. haematobium* infection (adjusted OR: 11.0, 95 % CI: 2.5–48.2), while having a urinary tract infection was significantly associated with *S. haematobium* infection (adjusted OR: 59.6, 95 % CI: 6.9–515.7). Risk factors for microhaematuria included living in rural areas (adjusted OR: 8.3, 95 % CI: 2.4–28.6) and *S. haematobium* infection (adjusted OR: 31.3, 95 % CI: 5.9–165.8).

**Conclusions:**

Our findings show that *S. haematobium* infection is hypoendemic in the Banfora municipality. However, the high prevalence of heavy infections is a particular concern, and targeted treatment strategies and complementary measures, including health education, should prioritize school-aged children living in rural areas.

## Graphical abstract

**Figure:**
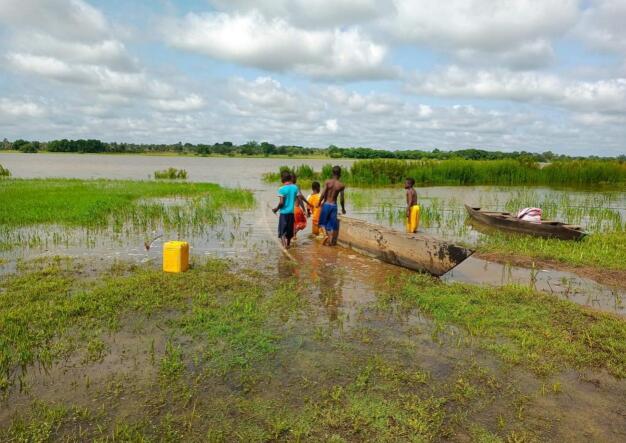

## 1 Introduction

Schistosomiasis is the second most common parasitic disease after malaria and the world's leading neglected tropical disease. It is estimated that schistosomiasis results in the loss of 2.5 million potential years of life and 24,000 deaths each year worldwide (WHO, 2020). Furthermore, over 90 % of the 240 million people affected by this poverty-related disease reside in sub-Saharan Africa, where *Schistosoma (S.) mansoni* and *S. haematobium* are the predominant species (WHO, 2020).

Urogenital schistosomiasis is caused by *S. haematobium*, with the main signs being macro- or microhaematuria alongside symptoms of a urinary tract infection (ANOFEL, 2021). Around 100 million people infected with *S. haematobium* suffer from haematuria and dysuria, as well as minor bladder morbidity. Meanwhile, more than 15 million people suffer from major bladder morbidity and kidney problems (WHO, 2019). Furthermore, children with chronic infections are at an increased risk of anaemia, stunted growth, cognitive impairment, and poorer academic performance (Freer et al., 2018).

In regional surveys conducted across different parts of Africa, the reported prevalence of urogenital schistosomiasis among school-age children has ranged widely, from 3.47 % to 52.7 % (Kapanga et al., 2022; Mushi et al., 2022), reflecting substantial local heterogeneity in transmission intensity and study focus. The main risk factors for *S. haematobium* infection were occupational and recreational contact with water (e.g. fishing, swimming, doing the laundry), knowledge and beliefs (e.g. level of education), socio-economic factors (e.g. income, parents' occupation), demographic factors (e.g. age, sex), and climatic and environmental factors (Ayabina et al., 2021).

Studies investigating the prevalence of microhaematuria in schoolchildren have mainly been conducted in East Africa, particularly in Ethiopia and Tanzania. The prevalence was found to be 22.54 % in Ethiopia (Deribew et al., 2022), ranging from 6.5 % to 46.2 % in Tanzania (Mushi et al., 2022; Knopp et al., 2018; Justine et al., 2024). However, the risk factors for microhaematuria are poorly documented (Deribew et al., 2022).

In Burkina Faso, schistosomiasis is focal, with *S. haematobium* and *S. mansoni* being the main species present in the country (Zongo et al., 2025). Owing to a sustained implementation of mass drug administration (MDA) using praziquantel from 2004, these parasites have been eliminated as a public health problem since 2013 (Ouedraogo et al., 2016). Nevertheless, there are still persistent schistosomiasis hotspots in some regions of the country (Zongo et al., 2025). The reasons for this include varying levels of drug effectiveness in resource-poor settings, as well as high rates of post-treatment reinfection in areas of high transmission (Hotez et al., 2023). The national prevalence of schistosomiasis among school-aged children has been estimated at 2.3 %, with the prevalence of *S. haematobium* infection reaching 5.8 % and 11.8 % in the Centre-Est and Sud-Ouest regions of the country, respectively (Zongo et al., 2025). The national prevalence of microhaematuria was 5.8 %, ranging from 6.9 % to 9.8 % in the Centre-Est and Sud-Ouest regions, respectively (Zongo et al., 2025). However, the latter study did not investigate the risk factors for *S. haematobium* infection and microhaematuria among school-aged children, and it was limited to certain regions. Consequently, the Cascades region, including the municipality of Banfora, was not included in the study (Zongo et al., 2025).

The environmental conditions in the Banfora municipality (including the Comoé River, ponds, lakes, and lowland rice fields) are conducive to the development and transmission of schistosomes, particularly *S. haematobium*. Indeed, in 1978, *S. haematobium* infection was highly prevalent in this municipality, affecting 20 % of school-age children in Banfora town and 86.7 % of those in the village of Tengréla. *S. mansoni* infection was also present in Tengréla, with a prevalence of 3.7 % (Boudin and Simonkovich, 1978). By 2008, Tengréla had only reported *S. haematobium*, with a prevalence of 13.3 % among 5–9 year olds and 23.3 % among 10–15 year olds (Zongo, 2010). The municipality is currently classified as a low-endemic schistosomiasis area (prevalence <10 %), and the last MDA for schistosomiasis took place in 2018. However, no assessment of the prevalence of schistosomiasis has been conducted since the last MDA. Furthermore, correlates of *S. haematobium* infection and microhaematuria remain unknown in this setting.

## 2 Materials and methods

### 2.1 Study design and area

**Fig. 1:**
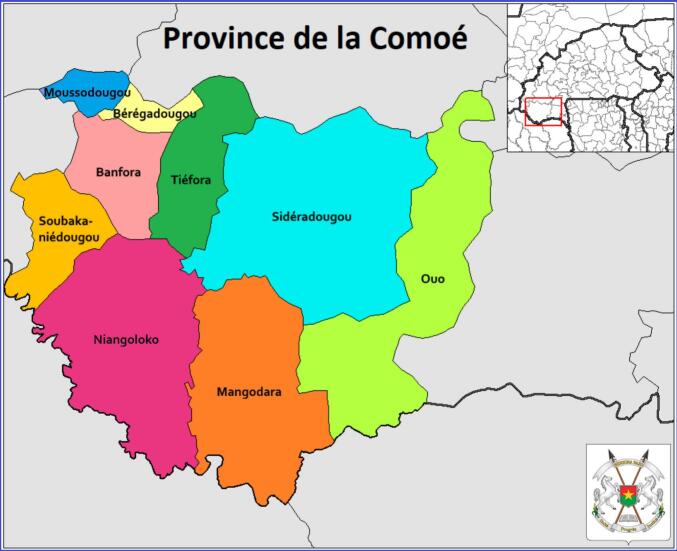
Study site. The Banfora municipality is highlighted in light pink. Source: https://gifex.com/fr/fichier/carte-de-la-province-de-la-comoe/ (For interpretation of the references to colour in this figure legend, the reader is referred to the web version of this article.)

This study was conducted in the municipality of Banfora, located in the south-west of Burkina Faso (Fig. 1). Banfora is the capital of the Cascades region and the Comoé province. It is located between latitudes 9°25′ and 10°37′ north and longitudes 3°50′ and 4°56′ west. The municipality covers an area of 934 km^2^ and comprises 15 sectors and 22 villages. In 2019, the municipality had 33,763 households and a population of 160,282 (INSD, 2020).

The Comoé River and its tributaries flow through the municipality, which is also home to the natural Lake Tengréla. These features create favourable conditions for fishing, rice cultivation and market gardening. This creates an environment suitable for the growth of the intermediate snail hosts of schistosomes, thereby increasing the risk of exposure to schistosomiasis. The main occupations are trade, rice cultivation, subsistence farming, and fishing.

Data collection was conducted in five public primary schools, two of which were located in urban areas and three in rural areas. The schools were selected according to the following criteria: a) location near a rice-growing area or a water point (such as a pond or lake); b) location around ten kilometers from the town of Banfora; and c) the school reachable by vehicle.

### 2.2 Study population

The study included schoolchildren from the five public primary schools in the Banfora municipality who met the following criteria: a) aged between 5 and 15 years; b) residents of the Banfora municipality for at least six months prior to the start of the study; c) provision of an assent by the child (if over 12 years of age); d) provision of a written informed consent by the child's parent or caregiver. Children were excluded if they: a) had taken praziquantel within the last six months; b) were unable to provide urine samples; or c) were absent on the day of the survey.

### 2.3 Sample size and sampling technique

The World Health Organization (WHO) recommends a minimum sample size of 50 school-age children for a baseline survey of helminth prevalence and intensity in schools (WHO, 2006). For practical reasons, however, we included 60 children in each selected school, giving a final sample size of 300 school-age children.

In each school, the lists of pupils in each class were compiled. Then, 10 pupils were randomly selected from each class, making a total of sixty children (there were six classes per school).

### 2.4 Field data and urine samples collection

Prior to the beginning of the survey, the selected schools were visited to explain the purpose of the survey to the teachers and the children's parents. Once the children's parents/caregivers had signed an informed consent form, a structured questionnaire was administered to the children and their parents. The questionnaire collected sociodemographic data, as well as information on the children's history of anti-helminthic treatment, general sanitation and hygiene practices and behaviours relating to contact with water. At each school, the children were given clean, dry, pre-labelled containers to collect freshly voided urine samples. These samples were collected on the day of inclusion between 10:00 a.m. and noon, after exercise.

The urine samples were partially processed for the detection of microhaematuria and leukocyturia in the laboratory at the Centre Médical Urbain de Banfora. They were then stored at −20 °C in the same laboratory before being transported in coolers to the laboratory of Parasitology-Mycology of Centre MURAZ for *S. haematobium* eggs screening.

### 2.5 Laboratory procedures

On the day of collection, all urine samples were visually examined for the presence of macrohaematuria. Microhaematuria and urinary tract infections were then detected using urine multistix reagent test strips (URIT Medical Electronic Co., Guangxi, China) following the manufacturer's instructions. The results were recorded as positive or negative.

A polycarbonate membrane filter with a pore size of 12 μm and a 10 mL plastic syringe were used to perform the urine filtration technique on each urine sample to detect *S. haematobium* eggs. Briefly, after thorough mixing, 10 mL of urine was drawn up in a syringe and gently pushed through the filter. The filter was then removed with forceps, placed on a clean glass microscope slide, stained with a drop of iodine, and examined under a 40× objective lens of a binocular microscope for *S. haematobium* eggs.

One slide was prepared for each urine sample and read by two independent biologists. A positive result was defined as the detection of at least one *S. haematobium* egg. The number of eggs found was counted, and the infection intensity was expressed as eggs per 10 mL of urine. Infection intensity was categorized as either light (1–49 eggs per 10 mL of urine) or heavy (≥ 50 eggs per 10 mL of urine) (Montresor et al., 1998).

### 2.6 Ethical considerations

This study was approved by the Comité d'éthique institutionnel de l'Institut National de Santé Publique (approval number 2024–0011/MSHP/SG/INSP/DG/CEI). All information relating to the study, including its potential benefits and risks, was recorded in an information form. This form was read out and explained to the participants' parents or caregivers. Children were only included in the study after written informed consent was obtained from their parents or caregivers. All information obtained from schoolchildren and their parents was treated as private and confidential, and records were stored in a locked cabinet. Children who tested positive for *S. haematobium* were treated with praziquantel (40 mg/Kg) free of charge.

### 2.7 Data analysis

The data were double-entered using Microsoft Excel 2016, then cleaned and analyzed using Stata 12 software (StataCorp, College Station, Texas, USA).

Descriptive statistics were used to summarize the data. The prevalence of *S. haematobium* infection was determined. Then, the prevalence of microhaematuria and urinary tract infection was calculated. The geometric mean intensity (GMI) of infection and its 95 % confidence interval (95 % CI) were estimated. Univariate and multivariate logistic regression analyses were conducted to identify factors associated with *S. haematobium* infection and microhaematuria (dependent variables). All independent variables with a *p*-value <0.20 in the univariate analysis were entered into the multivariate model. An automatic backward stepwise logistic regression approach was applied, in which variables were sequentially removed based on the Wald test, using an exclusion criterion of *p* > 0.05. Only variables that remained significant at *p* < 0.05 were retained in the final model. The strength of association was expressed as odds ratios (ORs) with corresponding 95 % confidence intervals (CIs).

## 3 Results

### 3.1 Socio-demographic characteristics and water contact behaviours of the study population

A total of 300 schoolchildren from five primary schools participated in the study, comprising 50.67 % girls (152/300) and 49.33 % boys (148/300). The mean age of the children was 8.79 ± 2.22 years, with over three-quarters belonging to the 5–10 age group (75.67 %). Most participants (64.67 %) lived within 1000 m of the water point and visited it (73.67 %) (Table 1).

**Table 1:** Socio-demographic characteristics and water contact behaviours of the schoolchildren (*n* = 300).

Variable	Frequency	%
Age group (years)		
5–10	227	75.67
˃ 10	73	24.33
Sex		
Female	152	50.67
Male	148	49.33
Residence		
Urban	114	38.00
Rural	186	62.00
Distance from the household to the water point		
˂ 500 m	83	27.67
500–1000 m	111	37.00
˃ 1000 m	106	35.33
Go to the water point		
No	79	26.33
Yes	221	73.67

### 3.2 Prevalence and intensity of *S. haematobium* infection among schoolchildren

Of the 300 schoolchildren tested, 11 (3.67 %) were infected with *S. haematobium* eggs. The GMI was 14.94 eggs/10 mL of urine (95 % CI: 4.96–44.98). Out of the 11 infected children, 8 (72.73 %) had a light infection while 3 (27.27 %) had a heavy infection.

### 3.3 Prevalence of macro- and microhaematuria, and urinary tract infection among schoolchildren

The prevalence of macrohaematuria and microhaematuria was 7.33 % (22/300) and 13.33 % (40/300), respectively. Similarly, the prevalence of urinary tract infection was 32 % (96/300) with a GMI of 55.71 leukocytes/μL (95 % CI: 43.14–71.93). Urinary tract infection was significantly more prevalent among girls (48.68 %) than among boys (14.86 %) (*P* < 0.001).

### 3.4 Correlates of *S. haematobium* infection

In univariable logistic regression analysis, children aged more than ten years were 4 times more likely to have *S. haematobium* infection than were those aged less than ten years (crude OR: 4.0, 95 % CI: 1.2–13.5). Urinary tract infection was significantly associated with *S. haematobium* infection (crude OR: 23.6, 95 % CI: 3.0–187.3). In multivariable logistic regression analysis, boys had higher odds of *S. haematobium* infection (adjusted OR: 11.0, 95 % CI: 2.5–48.2) than girls, and urinary tract infection remained significantly associated with *S. haematobium* infection (adjusted OR: 59.6, 95 % CI: 6.9–515.7) (Table 2).

**Table 2:** Univariate and multivariate analyses of factors associated with *S. haematobium* infection among schoolchildren (n = 300).

Variables	*S. haematobium* infection		COR (95 % CI)	AOR (95 % CI)	*P* value
	Yes (%)	No (%)			
**Age group (years)**					
5–10	5 (2.20)	222 (97.80)	1		
˃ 10	6 (8.22)	67 (91.78)	4.0 (1.2–13.5)		
**Sex**					0.001
Female	3 (1.97)	149 (98.03)	1	1	
Male	8 (5.41)	140 (94.59)	2.84 (0.7–10.9)	11.0 (2.5–48.2)	
**Residence**					
Urban	1 (0.88)	113 (99.12)	1		
Rural	10 (5.38)	176 (94.62)	6.4 (0.8–50.8)		
**Distance from the household to the water point**					
˂ 500 m	3 (3.61)	80 (96.39)	1.3 (0.3–6.5)		
500–1000 m	5 (4.50)	106 (95.50)	1.6 (0.4–7.0)		
˃ 1000 m	3 (2.83)	103 (97.17)	1		
**Go to the water point**					
No	2 (2.53)	77 (97.47)	1		
Yes	9 (4.07)	212 (95.93)	1.6 (0.3–7.7)		
**Urinary tract infection**					˂ 0.001
No	1 (0.49)	203 (99.51)	1	1	
Yes	10 (10.42)	86 (89.58)	23.6 (3.0–187.3)	59.6 (6.9–515.7)	

COR: crude odds ratio; AOR: adjusted odds ratio.

### 3.5 Correlates of microhaematuria

In univariable logistic regression analysis, both the child's residence and *S. haematobium* infection were found to be significantly associated with microhaematuria. Multivariable logistic regression analysis revealed that children living in rural areas were more likely to have microhaematuria than those living in urban areas (adjusted OR: 8.3, 95 % CI: 2.4–28.6). Similarly, participants infected with *S. haematobium* were more likely to have microhaematuria (adjusted OR: 31.3, 95 % CI: 5.9–165.8) than those who were uninfected (Table 3).

**Table 3:** Univariate and multivariate analyses of risk factors for micro-haematuria among schoolchildren (n = 300).

Variables	Micro-haematuria		COR (95 % CI)	AOR (95 % CI)	*P* value
	Yes (%)	No (%)			
**Age group (years)**					
5–10	30 (13.22)	197 (86.78)	1		
˃ 10	10 (13.70)	63 (86.30)	1.0 (0.5–2.3)		
**Sex**					
Female	22 (14.47)	130 (85.53)	1		
Male	18 (12.16)	130 (87.84)	0.8 (0.4–1.6)		
**Residence**					0.001
Urban	3 (2.63)	111 (97.37)	1	1	
Rural	37 (19.89)	149 (80.11)	9.2 (2.8–30.6)	8.3 (2.4–28.6)	
**Distance from the household to the water point**					
˂ 500 m	12 (14.46)	71 (85.54)	1.5 (0.6–3.5)		
500–1000 m	17 (15.32)	94 (84.68)	1.6 (0.7–3.5)		
˃ 1000 m	11 (10.38)	95 (89.62)	1		
**Go to the water point**					
No	9 (11.39)	70 (88.61)	1		
Yes	31 (14.03)	190 (85.97)	1.3 (0.6–2.8)		
***S. haematobium* infection**					˂ 0.001
No	31 (10,73)	258 (89,27)	1	1	
Yes	9 (81,82)	2 (18,18)	37.5 (7.7–181.2)	31.3 (5.9–165.8)	
**Urinary tract infection**					
No	23 (11.27)	181 (88.73)	1		
Yes	17 (17.71)	79 (82.29)	1.7 (0.9–3.3)		

COR: crude odds ratio; AOR: adjusted odds ratio.

## 4 Discussion

In our study, the overall prevalence of *S. haematobium* infection among schoolchildren in the Banfora municipality was 3.67 %, which can be categorized as low based on the WHO categories for endemic communities (WHO, 2020). This is consistent with previous reports from the Cascades region (Ouedraogo et al., 2016) and with the recently reported national prevalence of *S. haematobium* infection among school-aged children in Burkina Faso [2.3 % (95 % CI: 2.0–2.7)] (Zongo et al., 2025). However, the prevalence observed in our study (3.67 %) was substantially lower than those reported in regional or community-based surveys from other West African countries, including Côte d'Ivoire (14 %) (Angora et al., 2019), Mauritania (25.8 %) (Nakatt et al., 2024), Mali (50.2 %) (Agniwo et al., 2023), and Nigeria (67.3 %) (Balogun et al., 2022). In East Africa, localized studies have recorded prevalence levels ranging from 5.4 % to 52.7 % (Mushi et al., 2022; Knopp et al., 2018; Justine et al., 2024). It should be noted that these estimates derive from specific geographic areas or transmission hotspots and therefore do not represent national or continental prevalence.These variations could be explained by ecological and seasonal characteristics favouring *S. haematobium* transmission, which differ between study sites (Moser et al., 2014), and by the number of urine samples collected for diagnosing individual infections. The low prevalence of *S. haematobium* infection in the present study could also be attributed to the positive impact of praziquantel chemotherapy campaigns conducted in the study area from 2004 to 2018 (Zongo et al., 2025; Ouedraogo et al., 2016).

The predominance of light infections in our study (72.73 %) is in line with recent reports from Burkina Faso (Zongo et al., 2025; Ouedraogo et al., 2016) and other parts of sub-Saharan Africa, where light-intensity infections remain common (Mushi et al., 2022; Deribew et al., 2022; Knopp et al., 2018; Justine et al., 2024; Nakatt et al., 2024; Agniwo et al., 2023). However, the proportion of heavy infections (27.27 %) exceeds the 1 % threshold set by the WHO for elimination of schistosomiasis as a public health problem (WHO, 2020). This indicates ongoing transmission despite long-term MDA efforts. In accordance with WHO recommendations, sustained MDA coverage, coupled with intensified surveillance, targeted treatment of high-risk groups, and complementary measures such as health education, improved sanitation, and snail control, will be essential to consolidate gains and move toward the 2030 elimination targets (WHO, 2020).

In the current study, boys were found to be 11 times more likely to be infected with *S. haematobium* than girls. One possible explanation for this is that boys were significantly more exposed to water bodies than girls in our study. Socio-cultural factors, such as males being mostly engaged in water-contact activities like swimming, bathing, fishing, farming, and watering cattle, could lead to higher exposure among males (Geleta et al., 2015). Our findings were in line with those reported in Benin (Ibikounlé et al., 2014), Mauritania (Gbalégba et al., 2017), and Ethiopia (Geleta et al., 2015). However, similar studies carried out in Ghana (Okanla et al., 2003) and Nigeria (Gyuse et al., 2010) showed higher infection rates among females than males. In these studies, girls are more likely than boys to engage in activities such as washing dishes and clothes, which increases their contact with water bodies (Okanla et al., 2003).

The association between urinary tract infection and urogenital schistosomiasis found in our study is supported by several reports in which bacteriuria was frequently associated with *S. haematobium* infection, with *Escherichia coli* being the most prevalent species (Ossai et al., 2014; Amoo et al., 2017; Nwachukwu et al., 2018; Kone et al., 2022). However, we were unable to identify the main bacterial species present in children infected with *S. haematobium* because of the diagnostic method used (urine strip test). Therefore, this issue should be addressed in future studies. There are several possible explanations for this association. Firstly, *S. haematobium* eggs are expelled into the urine during an infection, and blood is also expelled from the bladder at the same time. This could encourage the growth of bacteria in the urinary tract, as blood is a potential culturing medium (Ossai et al., 2014). Secondly, bleeding could result from the migration of the spined eggs of *S. haematobium*. These torn surfaces then bleed, releasing blood for microbial utilization, and providing sites for microbial attachment and proliferation (Uwaezuoke et al., 2008). Thirdly, the association between bacterial infections and urogenital schistosomiasis may be due to either the sequestration of the bacteria in the parasite's skin (Hsiao et al., 2016), or to bacteria colonizing the worm's caecum (Ottens and Dickerson, 1972).

The prevalence of microhaematuria in our study (13.33 %) was higher than those reported in Burkina Faso (5.8 %) (Zongo et al., 2025) and Tanzania (2018) (6.5 %) (Knopp et al., 2018), but lower than those observed by other authors in Tanzania in 2022 (46.2 %) (Mushi et al., 2022) and in 2024 (18.5 %) (Justine et al., 2024), and elsewhere in Ethiopia (22.5 %) (Deribew et al., 2022). These differences could be explained by variations in the level of endemicity of urogenital schistosomiasis depending on the study sites.

In the present study, the prevalence of urogenital schistosomiasis detected using urine strips (13.33 %) was higher than that detected using the urine filtration method (3.67 %). Similar results have been reported in Burkina Faso (Zongo et al., 2025) and other African countries (Mushi et al., 2022; Deribew et al., 2022; Knopp et al., 2018; Justine et al., 2024) using urine strips. This observation could be attributed to the low sensitivity of the urine filtration method in diagnosing urogenital schistosomiasis, particularly in cases of light infection (≤ 5 eggs/10 mL urine) (Knopp et al., 2018), which subsequently could have led to an underestimation of the true prevalence of the disease. Indeed, in our study, most of the urogenital schistosomiasis cases (72.73 %) were of light infection. Therefore, highly sensitive techniques such as polymerase chain reaction (PCR) should be used in future studies to detect *S. haematobium* infection (ten Hove et al., 2008).

The increased risk of microhaematuria observed among children with *S. haematobium* infection in our study agrees with previous findings (Deribew et al., 2022; Knopp et al., 2018). Microhaematuria can be caused by several factors other than urogenital schistosomiasis, including genitourinary conditions such as urinary tract infections, kidney and bladder stones, urethral strictures, renal cancer, menstruation, and the intake of certain medications (Deribew et al., 2022). However, we found no such association with urinary tract infections in our study, nor did we assess other potential causes of microhaematuria.

Our study showed that living in rural areas is a risk factor for microhaematuria, as reported by Mazigo et al. in Tanzania (Mazigo et al., 2022). It was also observed that the two schools closest to Tengréla's lake (i.e. Tengréla A and Nékanklou A), recorded the highest prevalence of microhaematuria: 26.67 % and 20 %, respectively. This is not surprising, given that previous studies conducted in 1978 (Boudin and Simonkovich, 1978) and 2008 (Zongo, 2010) had shown that schistosomiasis is endemic around Tengréla Lake, a permanent body of water. Consequently, children living around the lake may experience continuous infection and rapid, frequent reinfection. Our findings highlight the importance of continuing deworming activities for the population living around Tengréla Lake, while raising awareness of schistosomiasis. In addition, malacological studies should be conducted to better assess the level of parasite transmission in the area.

This study has a number of limitations. Firstly, collecting a single urine sample from each schoolchild may have resulted in an underestimation of the prevalence and intensity of urogenital schistosomiasis, given that *S. haematobium* egg excretion varies from day to day in the same individual. Secondly, urine filtration is not very sensitive in diagnosing urogenital schistosomiasis, particularly in cases of light infection (Knopp et al., 2018). This may also have contributed to an underestimation of the true prevalence and intensity of the disease. Finally, our data were collected in November, a period of low parasite transmission.

## 5 Conclusion

This study presents an updated assessment of *S. haematobium* epidemiology after several decades of MDA in the study area. The generally low prevalence observed indicates that long-term MDA efforts have significantly reduced transmission and disease burden. Nonetheless, the persistence of infection in certain localities points to ongoing focal transmission and underscores the need for continuous surveillance and targeted interventions.

Consistent with the WHO guidelines, our findings highlight the importance of sustaining high MDA coverage while reinforcing integrated control measures such as improved water supply, sanitation, hygiene promotion, and environmental snail control. Strengthening these complementary strategies will be vital to consolidate the progress achieved through MDA and to move closer to the WHO 2030 target of eliminating schistosomiasis as a public health problem.

Ongoing monitoring of infection trends, coupled with locally adapted and evidence-based approaches, remains essential to maintain control and prevent resurgence in post-MDA settings.

## CRediT authorship contribution statement

**Mamoudou Cissé:** Writing – review & editing, Writing – original draft, Visualization, Validation, Supervision, Software, Resources, Project administration, Methodology, Investigation, Funding acquisition, Formal analysis, Data curation, Conceptualization. **Alamissa Soulama:** Writing – review & editing, Methodology, Investigation. **Constant Sirima:** Writing – review & editing, Methodology, Investigation. **Arthur D. Djibougou:** Writing – review & editing, Methodology, Investigation. **Souleymane Gnissi:** Writing – review & editing, Investigation. **Seydou Nakanabo-Diallo:** Writing – review & editing. **Muhammed Afolabi:** Writing – review & editing. **Issaka Zongo:** Writing – review & editing, Methodology.

## Ethical statements

This study was approved by the Comité d'éthique institutionnel de l'Institut National de Santé Publique (approval number 2024–0011/MSHP/SG/INSP/DG/CEI). Written informed consent was obtained from all study participants.

## Funding

Field data collection and lab work were supported by funds from a World Bank African Centres of Excellence grant (ACE02-WACCBIP) as part of a regional partnership between Centre MURAZ and the 10.13039/501100005601University of Ghana. The funder had no role in study design, data collection and analysis, decision to publish, or preparation of the manuscript.

## Declaration of competing interest

The authors declare that they have no competing interests.

## Data Availability

Data is contained within the article.
